# Association between supplementary private health insurance and visits to physician offices versus hospital outpatient departments among adults with diabetes in the universal public insurance system

**DOI:** 10.1371/journal.pone.0192205

**Published:** 2018-04-13

**Authors:** Chang Hoon You, Ji Heon Choi, Sungwook Kang, Eun-Hwan Oh, Young Dae Kwon

**Affiliations:** 1 Graduate School of Healthcare Management and Policy, The Catholic University of Korea, Seoul, Korea; 2 International Institute of Health, Seoul, Korea; 3 Department of Public Health, Daegu Haany University, Gyeongsan, Korea; 4 Department of Healthcare Management, Hyupsung University, Hwaseong, Korea; 5 Department of Humanities and Social Medicine, College of Medicine and Catholic Institute for Healthcare Management, The Catholic University of Korea, Seoul, Korea; Public Library of Science, UNITED KINGDOM

## Abstract

**Background:**

Diabetes mellitus is a chronic disease with a high prevalence across the world as well as in South Korea. Most cases of diabetes can be adequately managed at physician offices, but many diabetes patients receive outpatient care at hospitals. This study examines the relationship between supplementary private health insurance (SPHI) ownership and the use of hospitals among diabetes outpatients within the universal public health insurance scheme.

**Methods:**

Data from the 2011 Korea Health Panel, a nationally representative sample of Korean individuals, was used. For the study, 6,379 visits for diabetes care were selected while controlling for clustered errors. Multiple logistic regression models were used to examine determinants of hospital outpatient services.

**Results:**

This study demonstrated that the variables of self-rated health status, comorbidity, unmet need, and alcohol consumption significantly correlated with the choice to use a hospital services. Patients with SPHI were more likely to use medical services at hospitals by 1.71 times (95% CI 1.068–2.740, *P* = 0.026) compared to patients without SPHI.

**Conclusions:**

It was confirmed that diabetic patients insured by SPHI had more use of hospital services than those who were not insured. People insured by SPHI seem to be more likely to use hospital services because SPHI lightens the economic burden of care.

## Introduction

Diabetes mellitus is a chronic disease with a high prevalence across the world. In South Korea, the prevalence rate of diabetes among adults aged 30 or over is 8.03% in 2013 [1], and the incidence is on the rise. As of 2013, diabetes is the fifth main cause of death in South Korea. Because diabetes causes a high socioeconomic burden, the importance of early diagnosis and continuous optimal management is becoming more widely understood. Most diabetes patients, except for those with serious cases or complications, can be managed at physician offices, which maximizes the efficiency of healthcare resources [2]. Therefore, since 2011, the South Korean government has been implementing a system that increases a patient’s coinsurance payment for pharmaceuticals when the patient uses a hospital for outpatient care for any of 52 illnesses, including diabetes, which can be suitably treated at primary healthcare facilities. It has been reported that the implementation of this system has somewhat mitigated the influx of patients with minor illnesses at hospitals [3]. Along with this, in 2012, the South Korean government has been implementing a system that encourages the use of physician offices for diabetes care by reducing the patient’s copayment and offering various support services.

In South Korea, all citizens (except for those with low income, who are provided with Medical Aid) are covered by National Health Insurance (NHI), a social insurance. However, despite the NHI, only 55.6% of total healthcare costs were funded by public sources in 2015, well below the average of 72.9% in OECD countries. Due to the burden of high personal costs caused by the low coverage of NHI and increased demand for high-quality services, many people are purchasing private health insurance (PHI) to supplement the NHI [4]. Health insurance is generally known to increase policyholder use of medical services [5, 6]. Because insurance lowers the price of medical services for the policyholder, it encourages medical utilization. Proof that people with PHI use medical services more than non-insured people can be observed across the healthcare systems of multiple countries [6–13]. A previous study has shown that PHI not only results in quantitative growth in the use of medical services, but also influences the insured individual’s choice of healthcare providers. In many European countries, PHI policyholders were significantly more likely to seek care from specialists than were non-insured people [9,14]. However, no previous study has provided definite evidence of effects of PHI on the use of different types of medical institutions in South Korea. We assume that diabetes patients with PHI are likely to visit hospitals rather than physician offices in South Korea where patients can freely choose healthcare facilities or healthcare providers, because they believe hospitals to be more trustworthy.

In South Korea, there is a three-tier healthcare provision system in place that consists of primary, secondary, and tertiary health facilities; patients are free to choose any primary and secondary levels of medical institutions for outpatient treatment, with patient's copayment higher in secondary healthcare facilities rather than primary healthcare facilities. Tertiary hospitals can be accessed only with a referral from primary or secondary healthcare facilities, except for a few special cases, e.g., childbirth, emergencies, dentistry, family medicine treatment, rehabilitation [15]. Due to this freedom of choice, many patients opt to receive outpatient care from hospitals or tertiary medical institutions despite having conditions that are treatable at physician offices. It is possible that the probability of patients arbitrarily choosing higher-level medical institutions is even higher in cases of chronic diseases that require frequent medical care than in cases of emergencies or acute illnesses.

The choice of medical institutions and healthcare providers is influenced by various demographic, social, and economic factors [16–19]. Income level and type of health insurance are the main economic factors. Generally, using higher-level healthcare facilities increases medical expenses and the financial burden on individuals compared to using primary healthcare facilities for the same disease [20]. Therefore, individual economic factors can influence patient selection of medical institutions. In South Korea, there is a universal public health insurance system, but it is also possible to subscribe to supplementary private health insurance (SPHI) which covers the copayment and non-covered services by the NHI. Because SPHI indemnify up to 90% of the patient’s share of medical costs, the SPHI-insured have better access to higher-level medical institutions, such as hospitals and tertiary healthcare facilities, than the non-insured [21]. This study aims to examine the relationship between SPHI ownership and visits to physician offices (primary healthcare facilities) versus hospital (secondary or tertiary healthcare facilities) outpatient departments among adults with diabetes, using nationally representative data.

## Material and methods

### Data

We used data from the 2011 Korea Health Panel (KHP), a nationally representative sample of Korean individuals and their families that include data on demographic and socio-economic status, subjective and objective health status, access to health care, and PHI status. The KHP used a stratified multistage probability sampling design according to region and residence in order to select nationwide subjects from the 2005 Korea Census. The KHP conducted face-to-face interviews with 7,866 households (24,616 individuals) until 2015, but only the data from 2008–2013 are available to the public. We used the 2011 data because sample retention was low in 2012 and 2013. For enrollment in the study, study samples were only extracted from KHP records of outpatient care for diabetes among adults (aged 20 and older) diagnosed with the disease (ICD-10 codes E10-E14). We excluded those younger than 20 because they are unlikely to have PHI by their own decision.

The KHP data have several advantages. For instance, the KHP provides information about medical utilization in the preceding year, along with visit-based data such as diagnosis of disease, treatments used, length of stay, medical costs, and types of medical institution. Another advantage of the KHP for our purposes is the inclusion of specific information about the PHI of the insured. The KHP also includes data on health conditions and health behaviors that affect the likelihood of using medical services.

KHP data are available to the public via a website (https://www.khp.re.kr:444/) without any restriction. The protocol for our study was reviewed and approved by the Institutional Review Board of The Catholic University of Korea (MC16EISI0080) with a waiver for written informed consent because the data were obtained from a public database and analyzed anonymously.

### Variables

The main dependent variable is whether adults with diabetes use outpatient services of physician offices (primary healthcare facilities) or hospitals (secondary or tertiary healthcare facilities). In KHP, medical institutions are separated into two types: physician offices and hospitals.

Explanatory variables were employed based on previous empirical studies and economic models, including socio-demographic, economic, and health-related characteristics. The socio-demographic variables used in this study were age, sex, marital status, education, and residency. The residence variable was coded as urban or rural to measure access to medical services. For economic characteristics, annual household income per capita, health insurance type, and SPHI status were assessed. Annual household income per capita is the sum of family members’ annual salaries adjusted by the number of family members. Household income per capita has a logarithmic transformation. Two binary variables were used for primary health insurance: NHI, the mandatory national public insurance, and Medical Aid, public social support for the poor. Participant SPHI status describes whether patients had PHI plans when they visited medical institutions.

To control for participants’ health status and behavior, which can affect health care utilization, we used self-rated health status, number of comorbidities, unmet need, revisits, and the health behaviors of smoking, drinking, and regular exercising. Self-rated health status is measured on a 100-point scale, with zero as the worst and 100 as the best health status. We recorded the number of comorbidities for each patient, but only if those conditions required care through medication or continual follow-up with a doctor for more than three months after diagnosis. We included the respondents’ unmet needs for medical services in order to control for accessibility. The self-reported unmet need for health care in the preceding 12 months was reported via a question asking whether they experienced a time when they needed health care services but were unable to use them. The revisit variable indicated whether patients had a first visit to a physician or follow-up visits for diabetes care. Among the health behavior variables, we defined smoking as currently smoking at least one cigarette per week. We classed respondents as drinkers if they reported drinking alcohol in the preceding month. Regular exercise indicated whether a respondent spent at least half an hour performing moderate or vigorous physical activity at least three times per week.

### Statistical analysis

We considered each visit for an outpatient service as health care utilization. We used simple frequencies to describe the characteristics of the visits based on physician service by SPHI status. We used Chi-square and ANOVA tests to determine the effects of socio-demographic characteristics, economic status, and health-related variables on the purchase of SPHI. We added simple frequencies and univariable tests by medical institution (physician office vs. hospital) in the same manner. Multiple logistic regression models were used to examine the determinants of choosing a medical institution by analyzing episodic data and controlling for clustered errors. Because some respondents had more than one physician visit, the statistical assumption of independence was violated. In the presence of clustered errors, ordinary linear regression estimates or logistic regression estimates are biased, and the standard error can be incorrect. To correct those issues, regression models that control for that type of error are highly recommended [22]. We applied cluster-robust standard errors for error correction. We present the odds ratios (ORs) with 95% confidence intervals (CIs) and estimates from logistic regression on why diabetes patients chose a physician office or hospital outpatient departments. Additionally, we conducted correlation analysis and multicollinearity test using variance inflation factor to examine the relationship between explanatory variables. There was no problem caused by the correlation between explanatory variables. All statistical tests were conducted using STATA 14 (Stata Corp, College Station, TX, USA).

## Results

We analyzed data from 6,379 visits following our sample criteria. Table 1 presents the characteristics of all visits according SPHI status in 2011. About 45.5% of the 6,379 visits were covered by an SPHI. We found a significant difference between males and females in SPHI status. Respondents with SPHI were younger, more highly educated, and had higher incomes than their counterparts without SPHI. The non-SPHI insured evaluated themselves as less healthy than did the privately insured respondents. With regard to health-related behaviors, the proportion of non-SPHI insured who did not exercise regularly was lower than that among the privately insured. Regarding selection of medical provider for diabetes care, 85.8% of the non-SPHI insured visited a physician office compared to 79.3% of the SPHI insured (Table 1).

**Table 1:** Characteristics of individuals by SPHI status.

	Non-SPHI (n = 3,475)		SPHI (n = 2,904)		Total (n = 6,379)		Chi/t	*P*
	N	%	N	%	N	%		
Sex							26.11	<.0001
Female	1,940	55.8	1,435	49.4	3,375	52.9		
Male	1,535	44.2	1,469	50.6	3,004	47.1		
Age (mean ± SD)	71.7 ± 10.1		62.1 ± 9.9		67.4 ± 11.1		38.21	<.0001
Marital status							260.18	<.0001
Married	2,310	66.5	2,412	83.1	4,722	74.0		
Unmarried	1,165	33.5	492	16.9	1,657	26.0		
Education							416.68	<.0001
Elementary	2,005	57.7	1,010	34.8	3,015	47.3		
Middle school	599	17.2	496	17.1	1,095	17.2		
High school	567	16.3	850	29.3	1,417	22.2		
College or higher	304	8.7	548	18.9	852	13.4		
Residence							14.58	0.0001
Rural	2,246	64.6	1,742	60.0	3,988	62.5		
Urban	1,229	35.4	1,162	40.0	2,391	37.5		
Health insurance type							255.53	<.0001
Medical Aid	618	17.8	139	4.8	757	11.9		
NHI	2,857	82.2	2,765	95.2	5,622	88.1		
Household income per capita* (mean ± SD)	822.4 ± 608.7		1,310.1 ± 986.1		1,044.2 ± 838.6		15.9	<.0001
Self-rated health status (mean ± SD)	60.3 ± 17.3		65.6 ± 14.9		62.8 ± 16.5		-12.55	<.0001
Number of comorbidities (mean ± SD)	4.2 ± 2.7		3.1 ± 2.7		3.7 ± 2.7		15.89	<.0001
Unmet need							43.68	<.0001
Yes	365	10.6	470	16.2	835	13.17		
No	3,080	89.4	2,426	83.8	5,506	86.83		
Revisit							8.2	0.0042
Yes	3,329	96.1	2,730	94.6	6,059	95.4		
No	136	3.9	157	5.4	293	4.6		
Smoking							7.38	0.0066
Yes	623	18.1	602	20.8	1,225	19.32		
No	2,822	81.9	2,294	79.2	5,116	80.68		
Drinking							132.26	<.0001
Yes	1,651	47.9	1,806	62.4	3,457	54.52		
No	1,794	52.1	1,090	37.6	2,884	45.48		
Regular exercise							71.05	<.0001
Yes	475	13.8	633	21.9	1,108	17.47		
No	2,970	86.2	2,263	78.1	5,233	82.53		
Medical institution							45.81	<.0001
Hospital	495	14.2	600	20.7	1,095	17.2		
Physician office	2,980	85.8	2,304	79.3	5,284	82.8		

Abbreviation: SPHI, supplementary private health insurance; SD, standard deviation; NHI, national health insurance

*unit: 10,000 Korean Won

Table 2 presents the characteristics of the visit sample by medical provider for diabetes care; 82.9% of the 6,379 visits were to physician offices, while 17.1% of visits went to the hospital for diabetes care. The proportion of young, highly educated, and urban residents among those who visited the hospital was higher than among those who visited a physician office. In the group who visited the hospital, income level, use of SPHI insurance, and use of Medical Aid were all higher than in the group who visited a physician office. Respondents who visited the hospital reported lower self-rated health status and higher number of comorbidities. The health-related behavior of drinking also correlated to the selection of a medical provider (Table 2).

**Table 2:** Characteristics of individuals in visit sample by medical institution.

	Physician office (n = 5,284)		Hospital (n = 1,095)		Total (n = 6,379)		chi/t	*p*
	N	%	N	%	N	%		
Sex							0.01	0.9122
Female	2,794	52.9	581	53.1	3,375	52.9		
Male	2,490	47.1	514	46.9	3,004	47.1		
Age (mean ± SD)	67.8 ± 10.9		65.2 ± 11.8		67.3 ± 11.1		7.03	<.0001
Marital status							8.53	0.0035
Married	3,950	74.7	772	70.5	4,722	74.1		
Unmarried	1,334	25.3	323	29.5	1,603	35.9		
Education							27.75	<.0001
Elementary	2,567	48.6	448	40.9	3,015	47.2		
Middle school	906	17.2	189	17.3	1,095	17.2		
High school	1,143	21.6	274	25.0	1,417	22.2		
College or higher	668	12.6	184	16.8	852	13.4		
Residence							55.46	<.0001
Rural	3,412	64.5	576	52.6	3,988	62.5		
Urban	1,872	35.5	519	47.4	2,391	37.5		
Health insurance type							17.77	<.0001
Medical Aid	586	11.1	171	15.6	757	11.9		
NHI	4,698	88.9	924	84.4	5,622	88.1		
SPHI							45.81	<.0001
Insured	2,340	43.6	600	54.8	2,904	45.5		
Non insured	2,980	56.4	495	45.2	3,475	54.5		
Household income per capita* (mean ± SD)	1,018.6 ± 845.5		1,168.7 ± 793.4		1,044.2 ± 838.6		-5.40	<.0001
Self-rated health status (mean ± SD)	62.4 ± 16.2		59.6 ± 17.1		62.7 ± 16.4		6.60	<.0001
Number of comorbidities (mean ± SD)	3.6 ± 2.7		4.1 ± 2.8		3.7 ± 2.7		-6.07	<.0001
Unmet need							62.99	<.0001
Yes	611	11.7	224	20.5	835	13.2		
No	4,641	88.3	865	79.5	5,506	86.8		
Revisit							8.20	0.0042
Yes	3,329	96.1	2,730	94.6	6,059	95.4		
No	136	3.9	157	5.4	293	4.6		
Smoking							0.41	0.5205
Yes	1,007	19.2	218	20.0	1,225	19.3		
No	4,245	80.8	871	80.0	5,116	80.7		
Drinking							15.94	<.0001
Yes	2,923	55.6	534	49.1	3,457	54.5		
No	2,329	44.4	555	50.9	2,884	45.5		
Regular exercise							0.53	0.4674
Yes	926	17.6	182	16.7	1,108	17.5		
No	4,326	82.4	907	83.3	5,233	82.5		

Abbreviation: SD, standard deviation; NHI, national health insurance; SPHI, supplementary private health insurance

*unit: 10,000 Korean Won

Table 3 shows the results of a multiple logistic regression model to determine when diabetes patients use the outpatient service in a hospital. SPHI status, self-rated health status, number of comorbidities, unmet need, and drinking were all statistically significant predictors of hospital utilization; no other variables had a significant association with hospital use. The SPHI insured were 1.711 times more likely to use the hospital than the non-SPHI insured (95% CI, 1.068–2.740; *P* = 0.026). The self-rated health status and comorbidity also correlated with use of the hospital for diabetes care (OR 0.987, *P* = 0.010; OR 1.119, *P* = 0.004, respectively). Respondents with unmet need were less likely to use the hospital than respondents with no unmet need (OR 0.671, *P* = 0.010). In terms of health-related behaviors, drinkers were less likely to visit the hospital for diabetes care than non-drinkers (OR 0.581, *P* = 0.018), but smoking and regular exercise were not related with selection of a medical institution (Table 3).

**Table 3:** Determinants for using hospital outpatient service among diabetes patients after adjusting for clustered error.

	Odds ratio	95% CI	*P-*value
Sex (ref = female)	1.172	0.648–2.120	0.599
Age	0.943	0.791–1.123	0.512
Age^2^	1.000	0.998–1.001	0.624
Marital status (ref = unmarried)	1.333	0.846–2.098	0.214
Education (ref = elementary school)			
Middle school	1.331	0.760–2.332	0.316
High school	1.372	0.769–2.448	0.284
College or higher	1.719	0.851–3.470	0.131
Residence (ref = rural)	1.484	0.983–2.241	0.060
Household income per capita (log)	1.409	0.869–2.284	0.164
Health insurance type (ref = MA)	0.724	0.359–1.460	0.195
SPHI (ref = no)	1.711	1.068–2.740	0.026
Self-rated health status	0.987	0.977–0.996	0.010
Number of comorbidities	1.119	1.036–1.208	0.004
Unmet need (ref = no)	0.671	0.377–0.610	0.010
Revisit (ref = no)	1.058	0.714–1.568	0.776
Smoking (ref = no)	1.034	0.727–1.471	0.851
Drinking (ref = no)	0.581	0.370–0.910	0.018
Regular exercise (ref = no)	0.978.	0.588–1.625	0.932
Number of observations	5,881		
-2 Log likelihood	-2,416.51		
Wald test	46.18 (*P* = 0.0003)		
Pseudo R^2^	0.0714		

Abbreviation: CI, confidence interval; MA, Medical Aid; ref, reference; SPHI, supplementary private health insurance

## Discussion

This study demonstrated that SPHI ownership was related to diabetes patients’ use of hospital outpatient departments. Specifically, diabetes patients insured by SPHI were 1.711 times more likely than non-SPHI insured patients to choose hospitals over physician offices for outpatient care. Despite the fact that diabetes is a chronic disease that can be managed appropriately at physician offices, and that it is more efficient to manage it at the primary level of the healthcare system, many patients use a higher-level of medical institution for outpatient care.

### Diabetes patients might acquire services at hospitals because they have complications or their disease severity is high [23]

Although we cannot completely exclude the influence of disease severity or the presence of complications on the choice to use higher-level medical institutions, we did include the number of comorbidities as an explanatory variable. A second reason for this high use of hospital services is based on patients’ general behavior in using medical institutions. In South Korea, patients show an extremely high preference for higher-level and large hospitals because they have a low level of trust in and satisfaction with physician offices and believe that hospitals are significantly more trustworthy and satisfactory [24]. The current system offers few limits to using higher-level medical institutions, which could also be one of the reasons patients seek hospital treatment instead of going to a physician office. Given that South Korean hospitals are concentrated in large cities, we attempted to control for the difference in physical accessibility to hospitals by including place of residence as an explanatory variable.

On the other hand, many people purchase SPHI, which offers financial incentives for subscribers to use hospitals and might diminish the effect of the 2012 Korean government implementation to encourage patients to use primary health facilities. In particular, PHI schemes using fee-for-service systems have spread rapidly since 2009 and indemnify up to 90% of the patient’s share of medical costs. Using hospitals incurs a larger copayment for patients than using primary healthcare facilities for the same medical service. The costs for services that are not covered by the NHI are also often higher in higher-level institutions than in primary facilities, which increase the overall burden of medical expenses [25]. However, SPHI does not currently differentiate by type of medical institution when compensating patients for their medical costs. Therefore, SPHI significantly lightens patients’ financial burden when using hospitals, which facilitates patient preference for hospitals.

It is well known that the burden of medical costs influences the selection of healthcare facilities or healthcare providers. It has been identified in some countries that high-income patients choose specialists, whereas low-income patients opt for general practitioners [26]. It is also well known that the type of health insurance affects patient choice of healthcare providers. In several European countries, PHI seems to have a significant association with or impact on the use of specialist services [14]. Given that each country has its own healthcare provision and medical security system, it is difficult to come to a definite conclusion about how or why PHI influences people’s choice of healthcare providers. In countries with an SPHI system, such as South Korea, Denmark, and the Republic of Ireland, the fact that PHI covers a substantial part of the patient’s share of medical copayment is thought to affect their choice of healthcare provider or medical institution.

Many empirical studies have shown that PHI tends to increase the subscriber’s use of medical treatment. In South Korea, it was confirmed that those who enrolled in PHI tend to use more outpatient services and spend more on medical expenditures than those who did not. [27,28]. Beyond confirming the possibility of an increase in overall medical utilization, there should also be an analysis of differences according to type of medical institution and healthcare provider. However, no previous study has provided definite evidence of the correlation between SPHI and the use of different types of medical institutions. Thus, our study is significant because we considered the influence of purchasing SPHI on the use of different types of medical institutions using one year of information about diabetes patients’ outpatient visits as the unit of analysis and examining whether individual patients used physician offices or hospital outpatient departments.

The general characteristics of PHI policy-holders show that people with a higher income and education level are more likely to purchase PHI than those with lower incomes and education level [29–31]. On the other hand, people with higher income and education level might also be more likely to demonstrate a stronger preference for hospitals. To control for those confounding variables, we included income and education level as individual variables. However, variables that are impossible to measure objectively, such as personal preferences in medical care, can affect both the purchase of PHI and the choice of health facilities. If people with a strong preference for hospitals also have a strong preference for PHI, the effect of PHI on the use of hospitals as examined in this study is probably overestimated. Overcoming that limitation (endogeneity) will require a study of causality using time series data rather than cross-sectional data. For example, a future study could consider the difference in use of hospitals before and after purchasing PHI. Because the results of this study are based on cross-sectional analysis, the causal interpretation of SPHI on hospital service use should be avoided. Regarding health behaviors, drinking correlated with the choice of medical institution for diabetes care, but smoking and regular exercise did not. However, additional research is needed about health behavior variables because this study did not consider frequency, quantity, and intensity of alcohol consumption, smoking, or exercise. Another possible variable is the disease itself; however, by targeting a specific disease, we effectively controlled that influence. Because diabetes is common, it was easy to acquire a large enough sample. Also, because diabetes can be effectively managed at primary level medical institutions, it is a suitable subject for this study because a minority of patients with diabetes needs to use hospitals. Future studies should consider the influence of PHI on use of hospitals in the treatment of other chronic diseases such as hypertension.

This study analyzed the association of SPHI and diabetes outpatients’ use of hospitals. This study demonstrated that SPHI is a factor to the choice of medical institutions in South Korea’s universal public insurance system. An economic incentive from SPHI was confirmed in diabetes patients’ use of hospital outpatient departments even though they could receive appropriate care in physician offices.
